# Decreased Na^+^/K^+^ ATPase Expression and Depolarized Cell Membrane in Neurons Differentiated from Chorea-Acanthocytosis Patients

**DOI:** 10.1038/s41598-020-64845-0

**Published:** 2020-05-21

**Authors:** Zohreh Hosseinzadeh, Stefan Hauser, Yogesh Singh, Lisann Pelzl, Stefanie Schuster, Yamini Sharma, Philip Höflinger, Nefeli Zacharopoulou, Christos Stournaras, Daniel L. Rathbun, Eberhart Zrenner, Ludger Schöls, Florian Lang

**Affiliations:** 1 Paul Flechsig Institute of Brain Research, University of Leipzig, Leipzig, Germany; 2 German Center for Neurodegenerative Diseases, Tübingen, Germany; 3 Medical Genetics and Applied Genomics, University of Tübingen, Tübingen, Germany; 4 Transfusion Medicine, Medical Faculty, Eberhard Karl University, Tübingen, Germany; 5 Department of Internal Medicine III, University of Tübingen, Tübingen, Germany; 6 Department of Biochemistry, University of Crete Medical School, Heraklion, Greece; 7 Department of Ophthalmology, University of Tübingen, Tübingen, Germany; 8 Department Ophthalmology, Bionics and Vision, Henry Ford Hospital, Henry Ford, United States; 9 Department of Neurology and Hertie Institute for Clinical Brain Research, University of Tübingen, Tübingen, Germany; 10 Department of Vegetative and Clinical Physiology, University of Tübingen, Tübingen, Germany

**Keywords:** Neurophysiology, Neurological disorders

## Abstract

Loss of function mutations of the chorein-encoding gene *VPS13A* lead to chorea-acanthocytosis (ChAc), a neurodegenerative disorder with accelerated suicidal neuronal cell death, which could be reversed by lithium. Chorein upregulates the serum and glucocorticoid inducible kinase SGK1. Targets of SGK1 include the Na^+^/K^+^-ATPase, a pump required for cell survival. To explore whether chorein-deficiency affects Na^+^/K^+^ pump capacity, cortical neurons were differentiated from iPSCs generated from fibroblasts of ChAc patients and healthy volunteers. Na^+^/K^+^ pump capacity was estimated from K^+^-induced whole cell outward current (pump capacity). As a result, the pump capacity was completely abolished in the presence of Na^+^/K^+^ pump-inhibitor ouabain (100 µM), was significantly smaller in ChAc neurons than in control neurons, and was significantly increased in ChAc neurons by lithium treatment (24 hours 2 mM). The effect of lithium was reversed by SGK1-inhibitor GSK650394 (24 h 10 µM). Transmembrane potential (V_m_) was significantly less negative in ChAc neurons than in control neurons, and was significantly increased in ChAc neurons by lithium treatment (2 mM, 24 hours). The effect of lithium on V_m_ was virtually abrogated by ouabain. Na^+^/K^+^ α1-subunit transcript levels and protein abundance were significantly lower in ChAc neurons than in control neurons, an effect reversed by lithium treatment (2 mM, 24 hours). In conclusion, consequences of chorein deficiency in ChAc include impaired Na^+^/K^+^ pump capacity.

## Introduction

The widely expressed^1–3^ phosphatidylinositol lipid binding protein^4^ chorein up-regulates phosphoinositide-3-kinase signalling and participates in the regulation of diverse cellular functions. Chorein-sensitive functions include actin polymerization and cell stiffness^2,3,5,6^, degranulation^3,5^, autophagy and cell survival^4,7–12^. Chorea-acanthocytosis (ChAc) is a rare hereditary disease caused by loss-of-function-mutations of the chorein encoding gene *VPS13A* (vacuolar protein sorting-associated protein 13A)^7,13^, leading to progressive autosomal recessive neurodegenerative disease characterized by severe pleotropic movement disorders, epilepsy, decline of cognitive functions, and variable erythrocyte acanthocytosis^4,7,11,14–22^. Eventually the neurodegeneration results in severe disability and early death^16^.

Mechanisms implicated in the impact of chorein on cell survival include upregulation of the Ca^2+^ release activated channel moiety ORAI1^9,23–25^, which accomplishes store-operated Ca^2+^ entry (SOCE)^26^ leading to transient increases of cytosolic Ca^2+^ activity ([Ca^2+^]_i_). Upon store depletion ORAI1 is activated by the Ca^2+^ sensing proteins STIM1 and/or STIM2^27–29^. Alterations of [Ca^2+^]_i_ participate in the regulation of cell survival^30,31^. ORAI1 and SOCE are decreased in fibroblasts and neurons of ChAc patients^23,24^. In several cell types they could be increased by lithium^23,24,32^, an effect supporting cell survival^23,24^. As a matter of fact, lithium supports survival of ChAc neurons^4,23^, and favourably influences the clinical course of neurodegenerative disease^33–35^.

The effects of chorein and of lithium on ORAI1 and SOCE involve serum and glucocorticoid inducible kinase-1 SGK1^23^, a kinase dependent on phosphoinositide-3-kinase and regulating multiple target proteins including diverse transport proteins^36,37^. Most importantly, SGK1 is a powerful regulator of the Na^+^/K^+^ pump^38^. The pump is responsible for Na^+^/K^+^ equilibrium maintenance across cell membranes and is essential for proper cell function^39^. Impaired Na^+^/K^+^ pump has been considered a cause of neuronal cell death^4,40–48^.

The present study explored whether chorein deficiency and lithium influence neuronal Na^+^/K^+^ pump capacity. To this end, skin fibroblasts from ChAc patients and age-matched healthy individuals were reprogrammed to induced pluripotent stem cells (iPSCs) and further differentiated to cortical neurons. In those cells Na^+^/K^+^ pump capacity was quantified by using whole cell patch clamp.

## Results

### Representative characterisation of differentiated cortical neurons

**Figure 1:**
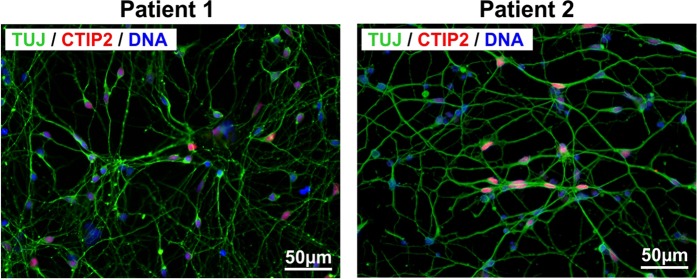
Characterisation of iPSC-derived cortical neurons. Patient-derived cortical neurons present typical neuronal morphology by expressing ß-III-tubulin (TUJ1, green) and the cortical layer V marker CTIP2 (red). Nuclei were counterstained with DAPI (blue). Scale bar = 50 µm.

To define the differentiation stage and cellular identity of generated iPSC-derived cortical neurons, cells were immuncytochemically analysed. A highly homogenous population of iPSC-derived cortical neurons could be detected by staining of neurons with ß-III-tubulin (TUJ, neuronal marker) and CTIP2 (cortical layer V marker) (Fig. 1).

### Sensitivity of Na+/K+ α1-subunit transcript levels and protein abundance to lithium treatment and SGK1-dependent regulation in healthy and ChAc neurons

**Figure 2:**
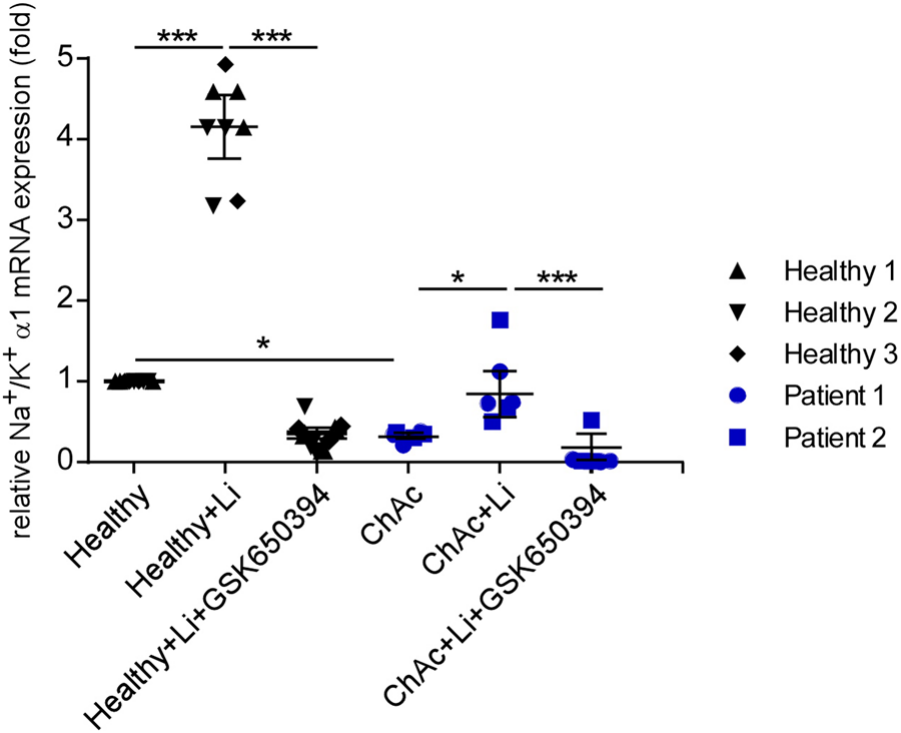
Effect of lithium on Na^+^/K^+^ α1-subunit transcript levels in neurons generated from healthy volunteers and ChAc patients in the absence or presence of SGK1 inhibitor GSK650394. Arithmetic means ± SEM (n = 6-9) of Na+/K+-ATPase transcript levels in neurons generated from healthy volunteers (black diamond, triangle, reverse triangle) and in neurons generated from ChAc patients (blue circle, square). Healthy and ChAc neurons were either untreated, pretreated with lithium (2 mM, 24h) or pretreated with lithium in the presence of SGK1 inhibitor GSK650394 (10 μM, 24h). Expression levels were normalized to the housekeeping gene GAPDH and relatively normalized to transcription value of Na+/K+ α1-subunit in control neurons. Data was derived from three independent culture experiments. (p < 0.05) and ***(p < 0.001) indicates significant difference, Dunn’s Multiple Comparison test.

**Figure 3:**
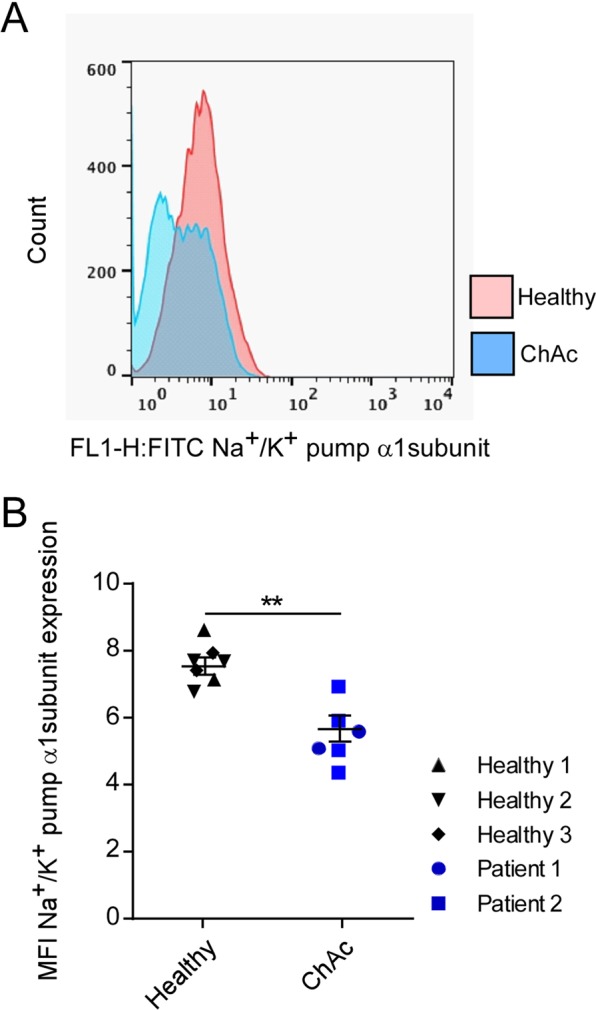
Na^+^/K^+^ α1-subunit protein levels in neurons generated from healthy volunteers and ChAc patients. **(A)** Original histogram of Na^+^/K^+^ α1-subunit protein abundance determined by flow cytometry in neurons generated from healthy volunteers (red) and ChAc patients (blue). **(B)** Means ± SEM (n  =  6-7) of Na^+^/K^+^ α1-subunit protein abundance (mean fluorescence intensity; MFI) determined by flow cytometry in neurons generated from healthy volunteers (black diamond, triangle, reverse triangle) and in neurons generated from ChAc patients (blue circle, square). Data was generated from two independent culture experiments. **(p  <  0.01) indicates significant difference between two groups, unpaired t-test.

Na^+^/K^+^ α1-subunit mRNA levels and protein abundance were determined using quantitative PCR and flow cytometry, respectively, in cortical neurons differentiated from induced pluripotent stem cells (iPSCs) of healthy individuals (control neurons) and patients with chorea-acanthocytosis (ChAc neurons). As shown in Figs. 2 and 3, the mRNA levels and protein expression of Na^+^/K^+^ α1-subunit were significantly lower in ChAc neurons than in neurons from healthy volunteers. Interestingly, the Na^+^/K^+^ α1-subunit transcript levels were significantly increased in ChAc neurons and neurons from healthy volunteers by treatment with lithium (2 mM, 24 h) (Fig. 2). In both, ChAc neurons and control neurons, the effect of lithium was abolished by inhibition of SGK1 by supplementation of GSK650394 (10 µM, 24 h).

### Effect of lithium treatment and SGK1 inhibitor on Na^+^/K^+^ pump capacity in ChAc and control neurons

**Figure 4:**
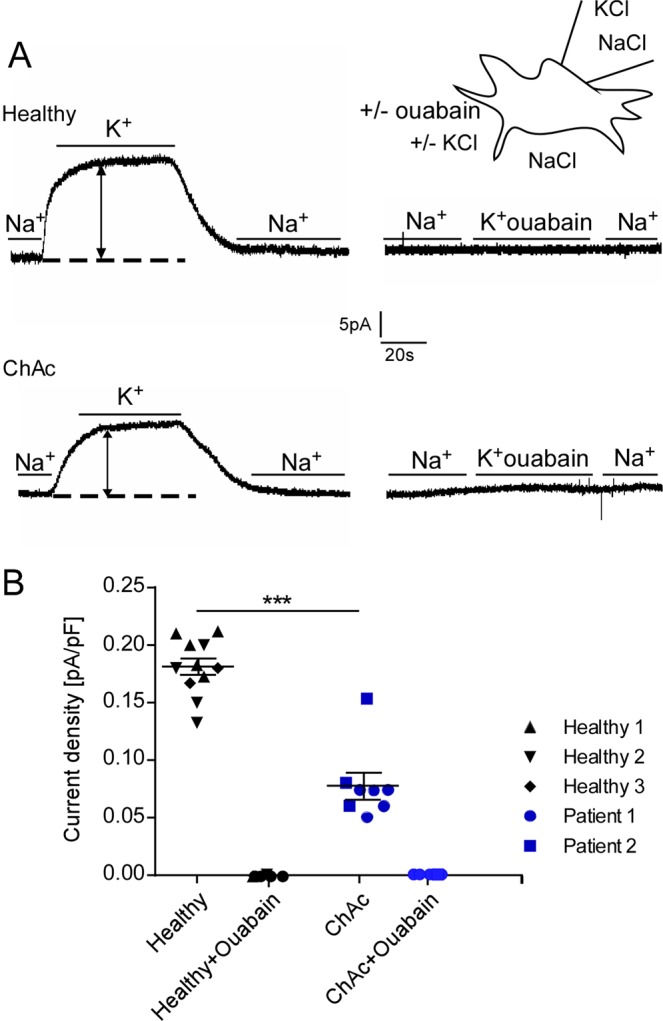
Effect of ouabain on Na^+^/K^+^ pump capacity in neurons generated from healthy volunteers and ChAc patients. **(A)** Original whole cell currents at −40mV cell membrane potential prior to, during and following an increase of extracellular K^+^ concentration from 0 to 5 mM in neurons generated from healthy volunteers (top) or ChAc patients (bottom) in the absence (left) and presence (right) of ouabain (100 µM). **(B)** Arithmetic means ± SEM (n = 6–11) of whole-cell current at −40mV normalized to cell capacitance in neurons generated from healthy volunteers (black diamond, triangle, reverse triangle) and in neurons generated from ChAc patients (blue circle, square) in the absence or presence of ouabain (100 µM). Data was derived from three independent culture experiments. **(p < 0.01) indicates significant difference, unpaired t-test.

In order to test whether Na^+^/K^+^ pump capacity is altered in ChAc neurons, whole cell patch clamp experiments were performed. Na^+^/K^+^ pump capacity was estimated from the outward current at -40 mV cell membrane potential induced by changing extracellular K^+^ concentration from 0 to 5 mM. As expected, the K^+^-induced current reflecting Na^+^/K^+^ pump capacity was completely abrogated in the presence of ouabain (100 µM) (Fig. 4). As illustrated in Fig. 4, the K^+^-induced current was significantly lower in ChAc neurons than in control neurons (Fig. 4B).

**Figure 5:**
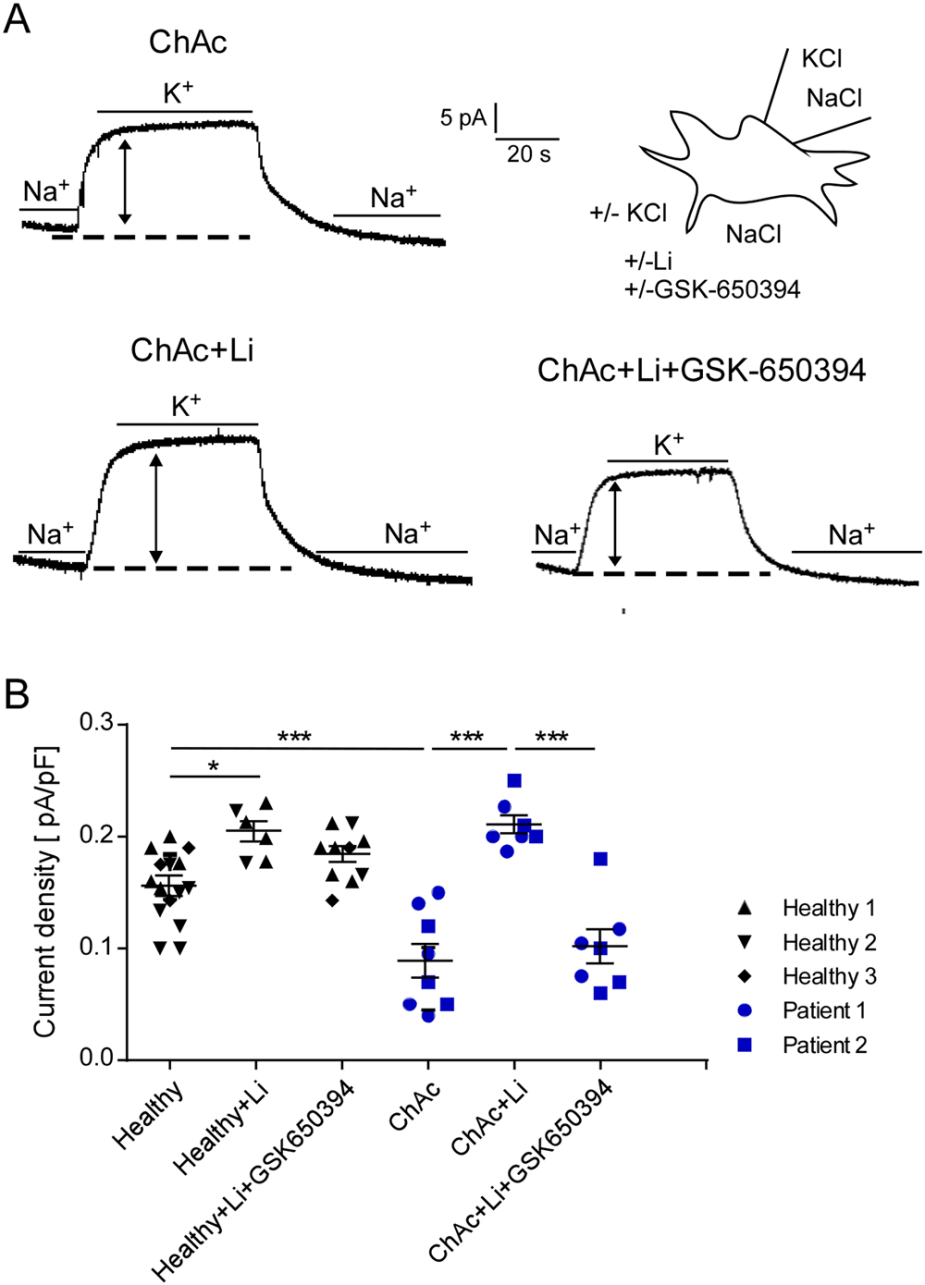
Effect of lithium on Na^+^/K^+^-ATPase capacity in neurons generated from ChAc patients in the absence and presence of SGK1 inhibitor GSK650394. **(A)** Original whole cell patch clamp by holding voltage at −40mV cell membrane potential prior to during and following an increase of extracellular K^+^ concentration from 0 to 5 mM in untreated ChAc neurons (upper row), lithium-treated (2 mM, 24 h) ChAc neurons (lower row, left) and lithium-treated ChAc neurons in presence of SGK1 inhibitor GSK650394 (10 µM, 24 h) (lower row, right). **(B)** Arithmetic means ± SEM (n = −15) of whole-cell current at −40mV normalized to cell capacitance in patient (blue circle, square) and control (black diamond, triangle, reverse triangle) neurons. Healthy and ChAc neurons were either untreated, pretreated with lithium (2 mM, 24 h) or pretreated with lithium in the presence of SGK1 inhibitor GSK650394 (10 µM, 24 h). Data was derived from three independent culture experiments. *(p < 0.05) and **(p < 0.01) indicates significant difference, Dunn’s Multiple Comparison test.

A second series of experiments explored whether the compromised Na^+^/K^+^ pump capacity in ChAc neurons can be increased by a 24 hours pretreatment with lithium (2 mM). As illustrated in Fig. 5, lithium pretreatment significantly increased the K^+^-induced current reflecting Na^+^/K^+^ pump capacity (Fig. 5A,B). Additional treatment with the SGK1 inhibitor GSK650394 (10 µM) decreased Na^+^/K^+^ capacity back to the level of untreated neurons (Fig. 5B).

### Effect of Na^+^/K^+^ pump, lithium and SGK1 inhibition on cell membrane potential in ChAc neurons

**Figure 6:**
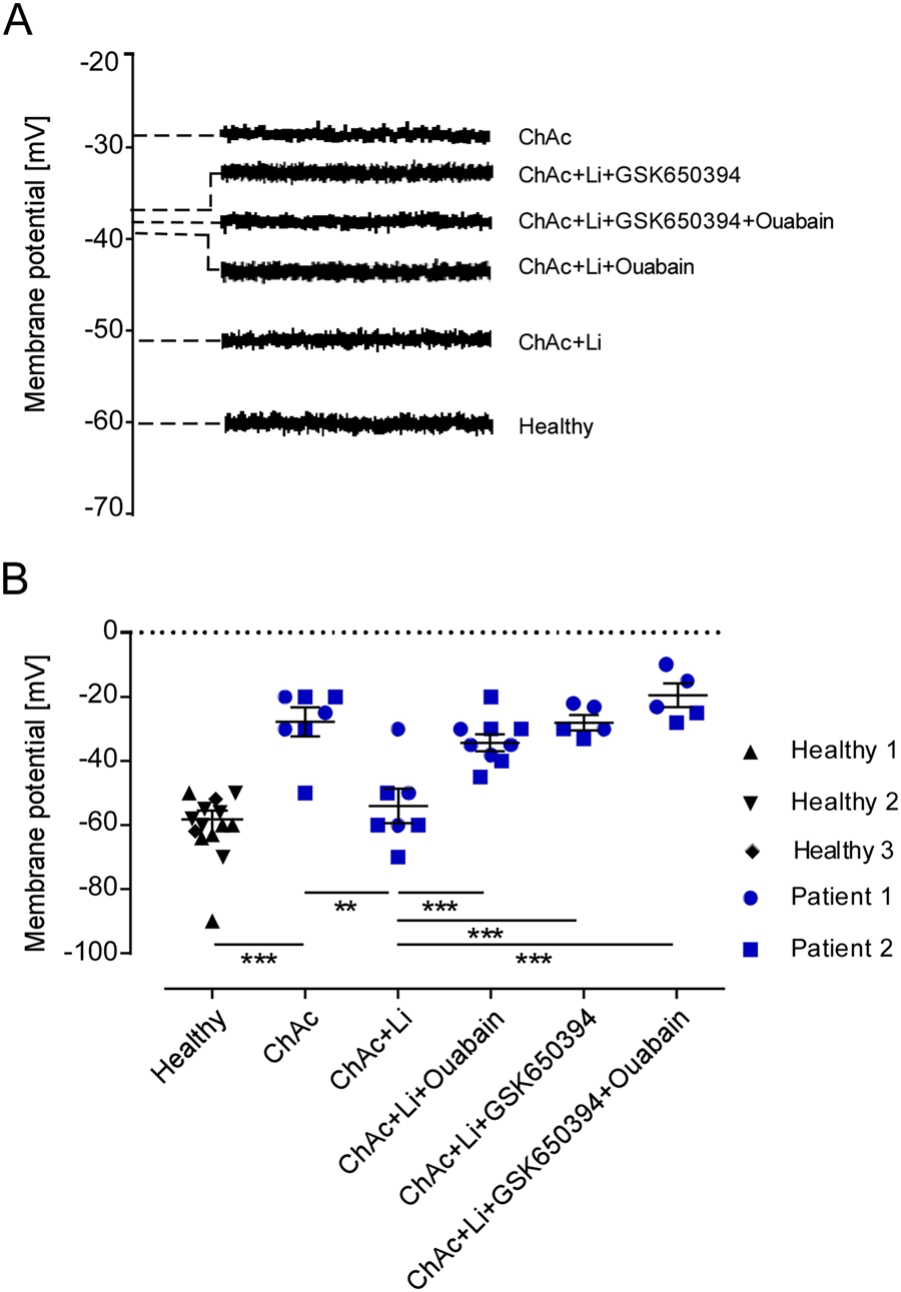
Effect of lithium on Cell membrane potential in neurons generated from healthy volunteers and ChAc patients in the absence and presence of ouabain and/or SGK1 inhibitor. **(A)** Original tracings of the cell membrane potential during current clamping at 0 A in untreated healthy and ChAc neurons and lithium-treated ChAc neurons (2 mM, 24 h) in presence/absence of SGK1 inhibitor GSK650394 (10 µM, 24 h) and/or oubain (100 µM, 24 h). **(B)** Arithmetic means ± SEM (n = 5–12) of the cell membrane potential during current clamping in neurons generated from healthy volunteers (black diamond, triangle, reverse) and in neurons generated from ChAc patients (blue circle, square). ChAc neurons were either untreated or pretreated with lithium (2 mM, 24 h) in the presence/absence of SGK1 inhibitor GSK650394 (10 µM, 24 h) and/or oubain (100 µM, 24 h). Data was derived from three independent culture experiments. **(p < 0.01), ***(p < 0.001) indicates significant difference, Dunn’s Multiple Comparison test.

The potential difference across the cell membrane was recorded in order to test whether the differences in Na^+^/K^+^ pump capacity between neurons generated from healthy volunteers and neurons generated from ChAc patients were paralleled by respective alterations of cell membrane potential. To this end, the current was clamped at 0 A. As illustrated in Fig. 6, the cell membrane potential was significantly less negative in ChAc neurons than in neurons derived from healthy volunteers. Lithium pretreatment significantly hyperpolarized ChAc neurons. As expected, ouabain (100 µM) depolarized the cell membrane (Fig. 6). The hyperpolarization of cell membrane potential of ChAc neurons by lithium treatment was disrupted by additional treatment with the SGK1 inhibitor GSK650394 (10 µM).

## Discussion

The results of this study uncover a novel functional consequence of defective chorein in chorea-acanthocytosis, i.e. impaired Na^+^/K^+^ pump capacity. The outward current induced by an increase of extracellular K^+^ concentration was significantly lower in patient neurons than in neurons differentiated from healthy volunteers. Lithium treatment of ChAc neurons reversed the reduction of the pump capacity. The effect of lithium was abrogated by pharmacological inhibition of SGK1. Along the same line, the cell membrane potential was less negative in ChAc neurons than in healthy neurons, and was hyperpolarized in ChAc neurons by lithium treatment.

The impairment of Na^+^/K^+^ ATPase expression and pump capacity in ChAc neurons and their up-regulation by a 24 hours pretreatment with lithium is reminiscent of the impaired expression of Ca^2+^ channel ORAI1 and thus store operated Ca^2+^ entry (SOCE) and their stimulation by lithium pretreatment^23^. The expression of Na^+^/K^+^ pump alpha subunit is enhanced following stimulation of Ca^2+^ entry with the Ca^2+^ ionophore ionomycin^49–52^. Possibly, the lithium sensitivity of Na^+^/K^+^ pump capacity is at least in part due to upregulation of SOCE. Conversely, Ca^2+^ entry through Ca^2+^ release activated Ca^2+^ channels is sensitive to cell membrane potential^53^ and is thus expected to be sensitive to Na^+^/K^+^ pump capacity. Both ORAI1^23^ and Na^+^/K^+^ pump activity^38^ are up-regulated by the serum/ glucocorticoid sensitive kinase SGK1.

Given the impact of Na^+^/K^+^ pump activity on neuronal cell survival^4,40–48^, the upregulation of Na^+^/K^+^ pump capacity by lithium could well contribute to the favourable effect of lithium on other neurodegenerative disorders including Alzheimer´s disease, Parkinson´s disease, Huntington´s chorea, amyotrophic lateral sclerosis or spinocerebellar ataxias^33–35,54,55^.

However, further mechanisms including upregulation of neurotrophic factors like BDNF, TrkB, and of Bcl-2 as well as upregulation of ORAI1^23^ have been invoked to participate in the effects of lithium. Lithium provides neurons protection against apoptosis through multiple mechanisms^56^ including downregulation of glycogen synthase kinase GSK-3β, the transcription factors p53 and FOXO3A, murine double minute (MDM), BAD and BAX, calpain, oxidative stress and of glutamate excitotoxicity^35,57,58^.

The reduced Na^+^/K^+^ pump capacity in ChAc neurons is paralleled by depolarization and the stimulation of Na^+^/K^+^ pump capacity by lithium is paralleled by hyperpolarization. The Na^+^/K^+^ pump modifies cell membrane potential by electrogenic transport^39^ and by enhancing K^+^ conductance of the cell membrane^59^. Given the impact of Na^+^/K^+^ pump capacity on cell membrane potential, the decreased Na^+^/K^+^ pump capacity could contribute to the decrease of cell membrane potential in ChAc neurons. The decreased cell membrane potential may foster excitation and thus contribute to the triggering of epileptic seizures in some ChAc patients^17–22^.

The present study addresses only the expression regulation of Na^+^/K^+^ pump α subunit 1. Neurons may, however, express not only Na^+^/K^+^ pump α subunit 1, but as well Na^+^/K^+^ pump α subunit 3^60^. Na^+^/K^+^-ATPase with α3-subunit has lower Na^+^ affinity and lower ouabain-sensitive transport activity than Na^+^/K^+^-ATPase with α1-subunit^61^. Na^+^/K^+^ α3 is highly expressed in neuronal branches^62^ as well as in dendritic spines^63^. Na^+^/K^+^ α3 extrudes intracellular sodium^64^, during intense neuronal activity^65^. Amyloid-beta in Alzheimer’s disease and alpha-synuclein in Parkinson’s disease interact with Na^+^/K^+^ α3 at the extracellular loop of the pump^66^.

The present study analyzed neuronal cells differentiated from iPSCs to cortical neurons. It should be kept in mind that those cells are not identical to neurons *in situ*. However, the target proteins of chorein and SGK1 including the Na^+^/K^+^ pump are presumably the same in cultured and *in situ* neurons. Given the wide expression of chorein^1–3^ and SGK1^36^, as well as the multiple targets of SGK1^36,37^, chorein- and SGK1- sensitivity of Na^+^/K^+^ pump capacity is expected to play a role in further cell types. Neurological symptoms are in ChAC patients presumably predominant due to the exquisite sensitivity of neuronal function to alterations of K^+^ gradients and potential difference across the cell membrane. It should further be taken into account that SGK1 regulates a variety of additional ion channels and transport proteins, which are likely to affect neuronal function.

In conclusion, Na^+^/K^+^ pump capacity is compromised due to a reduced expression of Na^+^/K^+^-ATPase in chorein deficient neurons derived from patients with chorea-acanthocytosis. This defect can partially be reversed by pretreatment with lithium in an SGK1-dependent manner. The observations provide additional insight into the cellular mechanisms underlying this severe neurodegenerative disease.

## Methods

### Generation of iPSCs

The study has been approved by the Ethical Commission of the University of Tübingen (598/2011) and experiments were conducted in accordance with German regulations and guidelines. Informed consent was signed from all participants and/or their legal guardian/s. Human dermal fibroblasts were isolated from ChAc patients (n = 2) and healthy volunteers (n = 3)^23,24^. ChAc patients carried two mutations in the *VPS13A* gene. Patient 1: c.[5761C > T], p.Arg1921*; c.[5761C > T], p.Arg1921* and Patient 2: c.[799C > T], p.Arg267*; c.[9109C > T], p.Arg3037*. Isolated dermal fibroblasts were cultivated in fibroblast cultivation medium (DMEM (Biochrom, Berlin, Germany), 10% fetal calf serum (FCS, Life Technologies, Thermo Fisher Scientific, Waltham, Massachusetts), 1% L-Glutamine (Biochrom)). The published protocol from Okita *et al*.^67^ was used for the generation of induced pluripotent stem cells (iPSCs). Briefly, fibroblasts were nucleofected (Nucleofector 2D, Lonza) with 1 μg of each plasmid (pCXLE-hUL, pCXLE-hSK and pCXLE-hOCT4) and further cultivated in fibroblast cultivation medium before adding 2 ng/ml FGF-2 (Peprotech) on day 2. The following day, medium was changed to Essential 8 (E8) medium containing 100 µM NaB (Sigma-Aldrich) and iPSC colonies were picked manually after 3-4 weeks. After further expansion on Matrigel coated 6-well plates using E8 medium, iPSCs were genomically and functionally analysed. This included the exclusion of plasmid-integration, SNP array analysis, and resequencing of mutation site, as well as proving the pluripotency by the expression of important pluripotency markers and the capacity to differentiate into cells of all three germ layers. For detailed methodological description see a previous publication^68^.

### Differentiation of iPSCs to neurons

Cortical neurons were differentiated from iPSCs as previously described^23,69^. In brief, neuronal differentiation was achieved by adding dual SMAD inhibitors (10 µM SB431542 (Sigma-Aldrich) and 500 nM LDN-193189 (Sigma-Aldrich)) to 3 N medium. At day 10, cells were collected, replated (ratio: 1:3) and further cultivated for 2 days in 3 N medium including 20 ng/ml FGF-2. Cells were further cultivated until day 27 in 3 N medium with medium change every other day. For the specific assays, cells were replated at the desired density (RNA/Protein isolation: 5×10^5^ cells per cm^2^; Patch clamp: 5 × 10^4^ per cm^2^) and further differentiated until day 37 to 41. To proof the identity of generated iPSC-derived cortical neurons, cells were immuncytochemically analysed using ß-III-tubulin (TUJ, neuronal marker) and CTIP2 (cortical layer V marker).

Where indicated, 2 mM lithium (Sigma-Aldrich) and/or 10 µM GSK650394 (Sigma-Aldrich) were added for 24 hours.

### Immunocytochemistry

iPSC-derived neurons were cultivated on 24-well plates on coverslips. Neurons were fixed in 4% paraformaldehyde (PFA) for 15 min. After washing 3 times with PBS, cells were permeabilized and blocked by incubation in blocking buffer (PBS, 5% BSA (Sigma Aldrich), 0.1% Triton X-100 (Sigma Aldrich)) for 45 min. Cells were stained with anti-ß-III-tubulin (TUJ, 1:1,000, T8660, Sigma Aldrich) and anti-CTIP2 (1:200, ab18465, Abcam) for 1 h followed by 3 times washing in PBS. Subsequently, cells were incubated with secondary antibodies (Alexa488 or Alexa568, 1:300, Life technologies) for 1 h. DAPI (1:10,000) was used to counterstain for nuclei by incubation for 15 min. Cells were embedded in Dako Mounting Medium (Dako) and images were acquired with Axio Imager Z1 (Zeiss).

### Quantitative Real-time PCR

PureLink™ RNA Mini Kit (Life Technologies, Germany) was used according to the manufacturer’s instructions for extracting total RNA^70^. For cDNA synthesis from total RNA, Superscript III cDNA Synthesis kit (Life Technologies, Germany) was utilized by following the manufacturer’s instructions. A total reaction mix volume of 10 µl was considered to set up the polymerase chain reaction (PCR) in order to determine the transcript levels of the respective cDNA, including 10 ng of cDNA, 250 nM forward and reverse primer and 2x qPCR Master Mix KAPA SYBR Green (PeqLab, Erlangen, Germany) according to the manufacturer’s protocol. The following cycling protocol was employed: initial denaturation at 95 °C for 3 min, 40 cycles of 95 °C for 10 sec, and 60 °C for 1 min. For the amplification the following primers were used (5′->3′orientation):

ATP1α1 F: AGCATCAATGACACAA; R: GCACTCTGCACCACTC and GAPDH; F: CGT CCC GTA GAC AAA ATG GT; R: TTG ATG GCA ACA ATC TCC AC.

By analysis of melting curves, PCR products were confirmed. CFX96 Real-Time System (Bio-Rad, Hercules, California) was used for real-time PCR amplifications. Real-time PCR experiments were performed in duplicate. Amplification of GAPDH was used as control in order to standardize the RNA content of all groups. To relatively quantify targeted cDNA (ATP1α1) expression, the ΔΔ^ct^ analysis method was applied as described earlier^71^.

### Flow cytometry

Protein abundance of Na^+^/K^+^ α1-subunit was characterised by an Na^+^/K^+^ ATPase a1-subunit antibody (3010, Cell signalling) and goat anti-rabbit IgG-FITC (554020, BD Pharmingen) in cortical neurons differentiated from iPSCs of healthy individuals and ChAc patients. Generated neurons were collected from the culture plate using Trypsin and centrifuged at 600 *g* for 5 minutes at room temperature, washed once with DPBS and fixed with 100 μl of fixation/permeabilization buffer (eBioscience) for 30 minutes in the dark followed by washing once with 1x permeabilization buffer (eBioscience). After washing, 0.5 μl primary antibody (anti-rabbit-Na^+^/K^+^ ATPase α1) in 50 μl permeabilization buffer was added. Neurons were incubated in the dark for 45 minutes. After washing cells twice with 1x permeabilization buffer 0.2 μl goat anti-rabbit IgG-FITC in 50 μl of 1x permeabilization buffer was added and incubated for another 30 minutes in the dark. Finally, neurons were washed twice with 200 μl DPBS. All washing steps were performed at 600 *g* for 5 minutes and room temperature. Neurons were acquired using BD FACSCalibur™ (BD Bioscience, Heidelberg, Germany) flow cytometry. Collected data of flow cytometry were analysed by FlowJo (BD). Na^+^/K^+^ α1-subunit protein expression was presented in mean fluorescence intensity (MFI).

### Patch clamp

Ouabain-sensitive K^+^-induced currents (I_pump_) indicating Na^+^/K^+^ pump capacity were measured by whole cell patch clamp at voltage-clamp mode recording at room temperature^72^. Neurons were continuously superfused at a rate of 200 ml/min through a flow system inserted into the dish. The bath was grounded via a bridge filled with the external solution. For manufacturing borosilicate glass pipettes (Harvard Apparatus, UK) with 2 to 4 MΩ resistance, a microprocessor-driven DMZ puller (Zeitz, Augsburg, Germany) was used in combination with a MS314 electrical micromanipulator (MW, Märzhäuser, Wetzlar, Germany). The I_pump_ were recorded by an EPC-9 amplifier (Heka, Lambrecht, Germany) at an acquisition frequency of 10 kHz and 3 kHz low-pass filtered and analyzed with Pulse fit software (Heka) and an ITC-16 Interface (Instrutech, Port Washington, NY)^72^.

To measure Na^+^/K^+^ pump capacity, K^+^-induced outward currents were recorded^72^ in the absence and presence of Na^+^/K^+^ pump inhibitor^72^ ouabain (100 µM) and/or antidepressant lithium^23,24^. The pipette solution contained (in mM): 30 NaCl, 20 KCl, 70 CsCl, 5 MgCl_2_, 5 HEPES, 5 Na_2_ATP and 5 ethylene glycol tetraacetic acid (EGTA). The external solution contained (in mM) 60 NaCl, 80 TEA-Cl, 1 MgCl_2_, 2.5 CaCl_2_, 5 NiCl_2_, 5 glucose, and 10 HEPES (pH 7.4, CsOH). By switching to a bath solution contained 60 NaCl, 80 TEA-Cl, 5 KCl, 1 MgCl_2_, 2.5 CaCl_2_, 5 NiCl_2_, 5 glucose, and 10 HEPES (pH 7.4, CsOH) I_pump_ were evoked. I_pump_ were measured at a holding voltage of -40 mV^73^. As the current rise time was dependent on rapidity of fluid exchange, the difference of steady state currents was evaluated.

For the membrane voltage recoding, after giga-Ohm seal formation (averaged seal resistance >1 GΩ) and entry into the whole-cell mode the current was clamped at 0 pA and the membrane voltage was continuously recorded. The pipette solution contained (in mM): 140 K-gluconate, 5 *N*-2-hydroxyethylpiperazine-*N*-2-ethanesulfonic acid (HEPES), 5 MgCl_2_, 1EGTA, 1 K-ATP, titrated with KOH to pH 7.4. Cells were superfused with NaCl solution containing in mM: 125 NaCl, 32 HEPES, 0 or 5 KCl, 5 glucose, 1 MgCl_2_, 1 CaCl_2_, and titrated with NaOH to pH 7.4.

### Statistics

Data of this paper are presented as arithmetic means ± SEM. Appropriate statistical analysis was applied to data including unpaired t-test or ANOVA and Dunn’s Multiple Comparison test as the post hoc analysis. Statistical significance was considered at p < 0.05.

### Ethical permission

The study has been approved by the Ethical Commission of the University of Tübingen (598/2011). Data were derived from independent culture experiments.
